# Supplementary material for: Conserving Marine Forests: Assessing the Effectiveness of a Marine Protected Area for Cystoseira sensu lato Populations in the Central Mediterranean Sea
Source: Plants (Basel). 2024 Jan 6;13(2):162. doi: 10.3390/plants13020162 (PMC10819874; doi:10.3390/plants13020162)

Conserving marine forests: assessing the effectiveness of a Marine Protected Area for *Cystoseira sensu lato* populations in the central Mediterranean Sea

Francesco Paolo Mancuso [1,2]*, Gianluca Sarà [1,2], Anna Maria Mannino A.M. [2,3]

[1]Department of Earth and Marine Sciences (DiSTeM), University of Palermo, viale delle Scienze Ed. 16, 90128 Palermo, Italy

[2]NBFC, National Biodiversity Future Center, Palermo 90133, Italy

[3]Department of Biological, Chemical and Pharmaceutical Sciences and Technologies, University of Palermo, 90123 Palermo, Italy

*Correspondence: francesco.mancuso@unipa.it; Tel.: +39 091 23860844

Supplementary materials

Table S1 - PERMANOVAs results of the structure and composition of the *Cystoseira s.l.* assemblage among the MPA's zones.

Source of variation		Structure				Composition			
	df	MS	Model-F	R2		MS	Model-F	R2	
zone	2	0.103	55.027	0.707	***	0.654	40.394	0.643	***
site (zone)	3	0.020	10.706	0.206	***	0.173	10.711	0.256	***
transect(site(zone))	6	0.002	1.237	0.048	ns	0.018	1.131	0.054	ns
Residuals	6	0.002		0.039		0.016		0.048	
Total	17			1.000				1.000	

Note:

Zone was fixed and orthogonal with two levels (zone A, zone B and zone C), site was random and nested in zone with two levels (1 and 2), while transect was random and nested in site and zone with 3 levels (1, 2 and 3). PERMANOVAs based on Bray–Curtis measure of square-root transformed abundances (structure) or Jaccard measure (species presence/absence data) of *Cystoseira s.l.* assemblage. P-values were calculated using 9.999 permutations. Signif. codes: *** $p < 0.001$, ns $p > 0.05$

Table S2 - PERMANOVAs results of the structure and composition of the *Cystoseira s.l.* assemblage among all areas investigated (MPA's zones and unprotected sites).

Source of variation		Structure				Composition			
	df	MS	Model-F	R2		MS	Model-F	R2	
area	4	0.104	68.019	0.818	***	0.755	57.042	0.798	***
site (area)	3	0.020	13.101	0.118	***	0.173	13.104	0.138	***

Source of variation		Structure				Composition			
	**df**	**MS**	**Model-F**	**R2**		**MS**	**Model-F**	**R2**	
transect(site(area))	8	0.003	1.640	0.039	ns	0.017	1.285	0.036	ns
Residuals	8	0.002		0.024		0.013		0.028	
Total	23			1.000				1.000	

Note:

Area was fixed and orthogonal with five levels (zone A, zone B, zone C, white and urban), site was random and nested in zone with one or two levels, while transect was random and nested in site and zone with 3 levels (1, 2 and 3). PERMANOVAs based on Bray–Curtis measure of square-root transformed abundances (structure) or Jaccard measure (species presence/absence data) of *Cystoseira s.l.* assemblage. P-values were calculated using 9.999 permutations. Signif. codes: *** $p < 0.001$, ns $p > 0.05$

Table S3 Average similarity of the *Cystoseira s.l.* assemblage between and within the different zones investigated.

Structure						Composition					
**Average**	Similarity	between/within	groups			Average	Similarity	between/within	groups		
	Zone B	Zone A	white	Zone C	urban		Zone B	Zone A	white	Zone C	urban
**Zone B**	84.961					Zone B	87.387				
**Zone A**	47.027	75.826				Zone A	50.869	76.864			
**white**	51.166	50.167	84.876			white	54.158	56.108	96.078		
**Zone C**	69.174	57.129	63.98	76.031		Zone C	80.524	58.397	67.48	86.007	
**urban**	20.577	29.601	34.561	28.46	87.241	urban	34.668	44.507	45.116	36.129	92.593

Figure S1 – Example of experimental design.

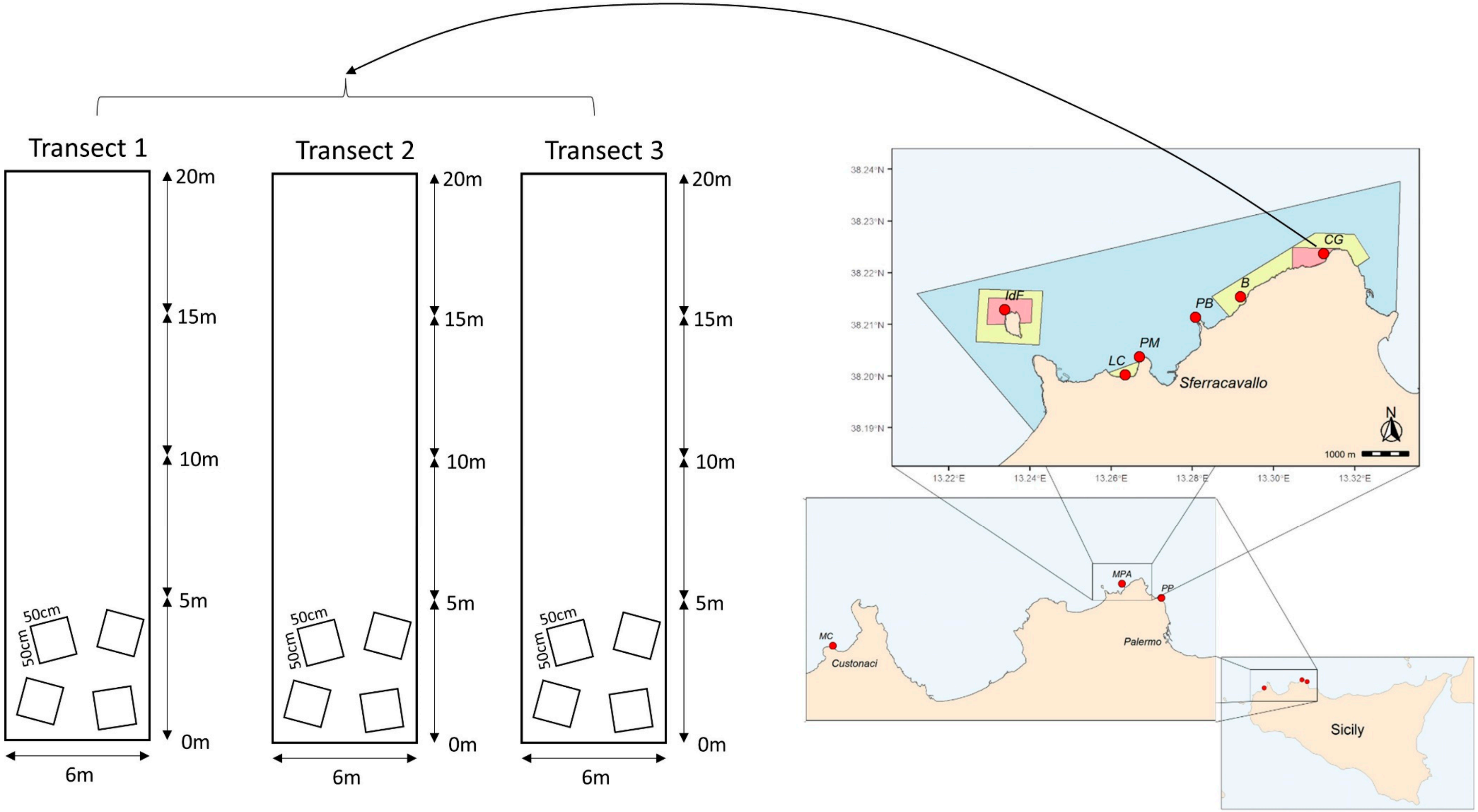

Supplement: Supplementary file 1 [file plants-13-00162-s001.zip › plants-2761118-supplementary.pdf]
